# Computed tomography scan measurements of the globe and orbit to assess the risks of traumatic complications from medial peribulbar anaesthesia

**DOI:** 10.1186/s12871-022-01633-5

**Published:** 2022-04-30

**Authors:** Jon Clarke, Huey Ming Seah, Aaron Foo, Marc Agzarian, Stewart Lake

**Affiliations:** 1 Department of Anaesthesia, Flinders Medical Centre, Adelaide, South Australia Australia; 2 South Australia Medical Imaging, Flinders Medical Centre, Adelaide, South Australia Australia; 3 Faculty of Medicine, Flinders University, Adelaide, South Australia Australia; 4 Department of Ophthalmology, Flinders Medical Centre, Adelaide, South Australia Australia

**Keywords:** Peribulbar anaesthesia, Computed tomography, Orbital anatomy

## Abstract

Complications of peribulbar anaesthesia include retrobulbar haemorrhage, globe perforation and brainstem anaesthesia. Therefore, this study took measurements relating the proximity of medial canthus to the optic nerve and also the safe angle between orbit and globe using 200 multiplanar reconstructed computed tomography (CT) images of the orbit. The principal results show that in 1.5% of the sample, the optic nerve is within 20 mm of the medial canthus, with a minimum distance of 15 mm. One% have a safe angle of 10 degrees or less between bone and globe. None of the demographic data, nor axial length were predictive of these results. We have shown that there are a minority of patients with unusual orbital anatomy. This places them at a theoretical higher risk of complications. These cases are not currently predicted by measured data.

## Introduction

Currently in Australia, the most common form of anaesthesia for cataract surgery is the peribulbar block [1]. One of the most common techniques uses 25 mm 25 gauge needle. The eye is held in neutral gaze and the needle is inserted at the medial canthus in a perpendicular plane. It is slowly advanced and if bony resistance is met then the needle is adjusted to a slightly more medial direction until the desired depth is reached. This is usually about 15 to 20 mm. Along this trajectory, there lies the potential to inadvertently penetrate the globe, damage the optic nerve and cause haemorrhage. The literature regularly contains reports including large case series [2] where the needle causes trauma to these structures. The reported incidence of globe perforation is around 1 in 5000 in large self-reported surveys [3, 4] and around 1 in 2000 to 4000 in single centre audits [5, 6]. The reported incidence of optic nerve sheath penetration in large self-reported surveys ranges from of 1 in 57,850 [3] and in single centre audits the range is 1 in 3000 to 6800 [5, 7]. The consequences of these complications are severe. Globe perforation may result in further surgery with loss of vision or blindness. Needle trauma to the optic nerve may result in loss of vision and brainstem anaesthesia. Brainstem anaesthesia is a spectrum of features including loss of vision in the contralateral eye, deafness, profound nausea through to unconsciousness and cardio-respiratory arrest [8]. The anaesthesia will work more rapidly and effectively when inserted more deeply into the orbit but this may increase the risk of complications. There is also an issue of terminology regarding peribulbar versus retrobulbar needle placement. Peribulbar anaesthesia aims to place the needle tip outside the muscle cone whereas retrobulbar is within. Anatomical study shows no evidence of an intermuscular septum separating an intraconal and an extraconal space. Instead describing a common spreading space, the corpus adiposum [9]. Furthermore, ultrasound study of a planned extraconal needle placement of a 25 mm needle demonstrated an actual 20% intraconal placement [10]. Therefore, peribulbar anaesthesia is only an intension of extraconal needle placement. The intraconal complications above are genuine risks.

Currently, the axial length is routinely measured for ophthalmic surgery.It is already known that there is some association between globe perforation and an axial length of 25 mm or greater [11].

There is one published report of actual optic nerve trauma from a peribulbar block. Distances can be measured but may not be representative of the general population [12].

MRI has been used to assess the risks of peribulbar anaesthesia to extra ocular muscles in the infero-temporal quadrant (myopic patients were excluded) [13].

Therefore, this study aimed to take a population sample representing cataract surgery patients. Then measure orbital anatomy relevant to medial peribulbar anaesthesia causing globe or optic nerve injury using CT scans. Currently, there is no data regarding the measurement of these parameters. Therefore we can assess the risk of these complications for any given needle insertion depth and angulation.

## Methods

This study was approved by the Southern Adelaide Clinical Human Research Ethics Committee (reference AUD/20/SAC/204).

### Study population

This was a single-centre retrospective study of patients over 60 years old between September and October 2020 who underwent multi-slice helical CT head studies for non-orbital related diseases or indications. The first consecutive 100 male and 100 female patients who underwent CT head studies were recruited into the study, allowing assessment of one orbit for a total of 200 orbits for analysis. Scans were only included if the patients had a neutral gaze where the whole length of the optic nerve and the lens were aligned. Patients were excluded if they had active or previous known orbital or ocular diseases or surgery.

### Image acquisition

All CT head studies were acquired using a Canon Aquilion ViSION 320 slice scanner (Canon Medical Systems, Otawara, Japan) or Philips Brilliance iCT 256 slices canner (Philips Healthcare, Eindhoven, Netherlands). Non-contrast or contrast-enhanced studies were accepted for the purpose of this study. The CT images were acquired in the axial plane. Scanning parameters on the Canon scanner are set at tube voltage of 120 kV, tube current of 378 mAs and slice thickness of 0.625 mm. Scanning parameters on the Philips scanner are set at tube voltage at 120 kV, tube current of 190 mAs and slice thickness of 0.4 mm. The thin slice data was reviewed using multi-planar reconstruction in the Picture Archive and Communication System (PACS) (Carestream VuePACS Version 12.2.1.0104, Carestream Health, Rochester, United States of America).

### Image interpretation

All images were reviewed on a dedicated workstation with diagnostic quality monitors (Barco Nio 3 megapixel colour, Barco, Kortrijk, Belgium). The images were reviewed in chronological order by a Radiology trainee and a medical student who had undertaken a Radiology rotation and confirmed by a Consultant Radiologist. The bony margins of the orbit were measured at a window width of 2000 Hounsfield Units (HU) and centre of 800 HU. The soft tissue structures were evaluated at a window width ranging from 600 to 800 HU, and centre of 0–100 HU. The images were windowed to a subjectively optimal contrast resolution for assessment.

Multiplanar reconstruction (MPR) of the CT images were performed with the axial axis aligned with the optic nerve and the lens, and the sagittal axis aligned with the midline. All measurements were performed on the MPR axial images on the slice where the optic nerve sheath of the assessed globe can be viewed in its entirety.

**Fig. 1:**
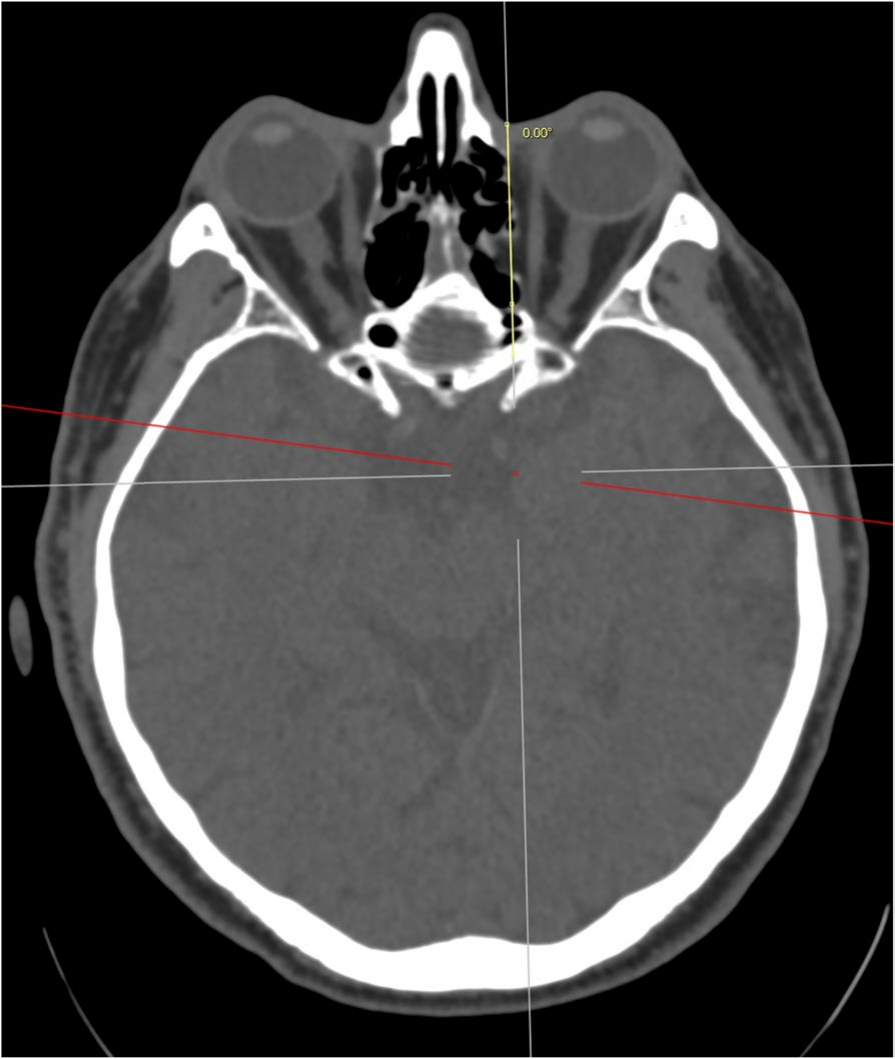
Reference line at medial canthus in the sagittal axis of the head

**Fig. 2:**
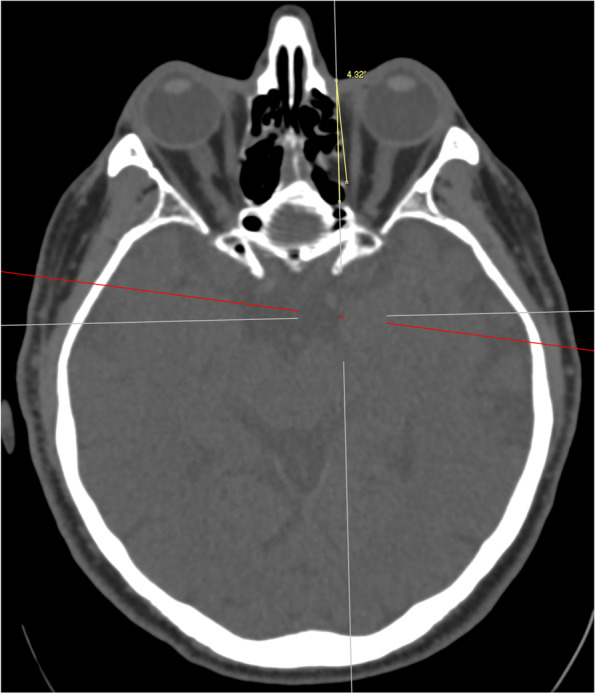
Angle between the reference line and the medial orbital wall (MOW)

**Fig. 3:**
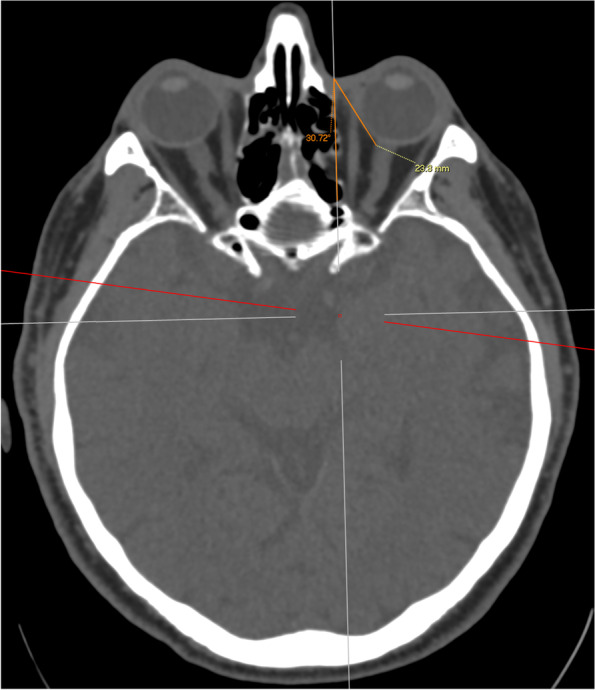
Angle between the reference line and the orbital tangent, and length of the orbital tangent from medial canthus to the optic nerve sheath

**Fig. 4:**
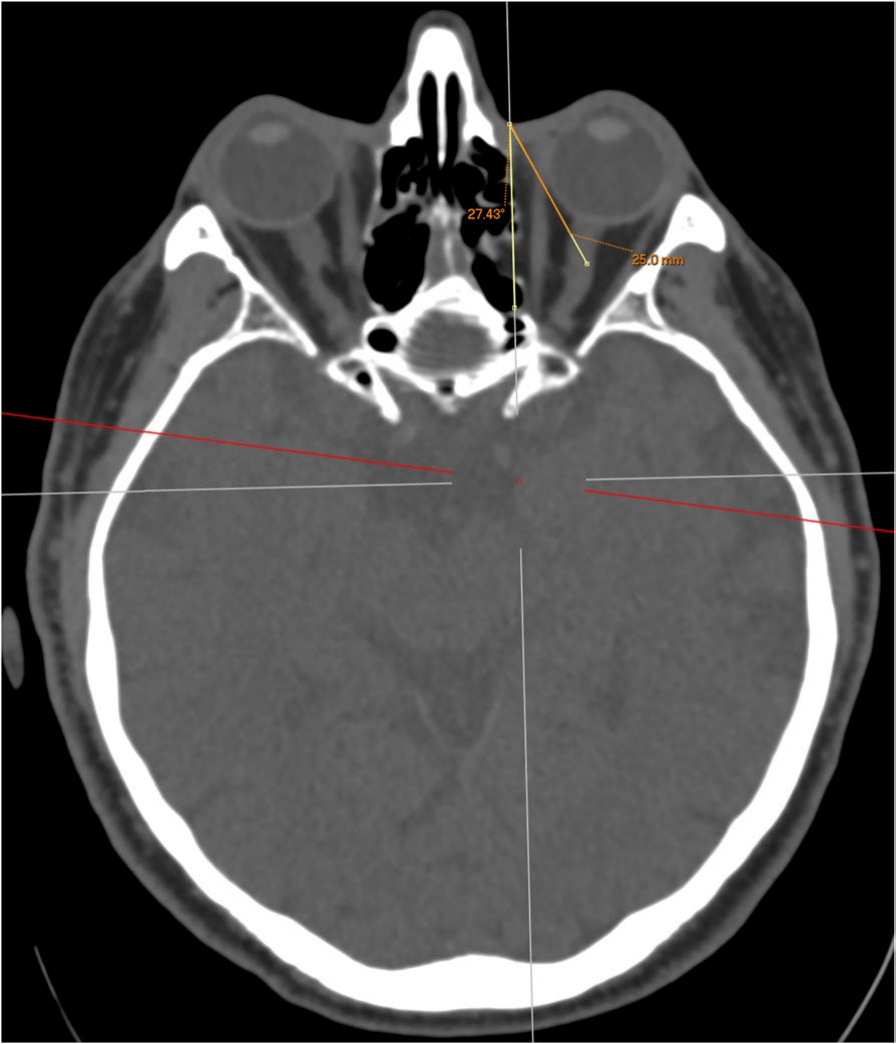
25 mm line drawn from the medial canthus to the optic nerve sheath and the corresponding angle with the reference line

**Fig. 5:**
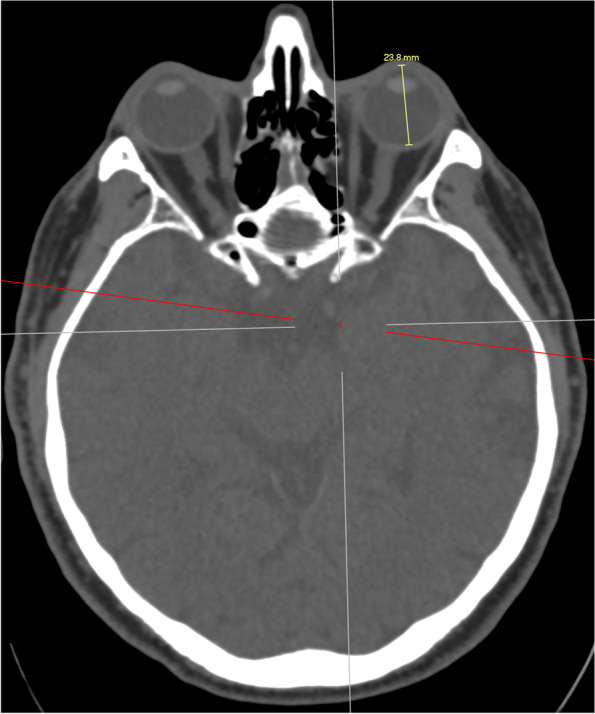
Length of orbital diameter (axial length)

A reference line was first drawn from the medial canthus extending from anterior to posterior along the sagittal axis of the head (Fig. 1). This is also of clinical importance as it represents the ideal trajectory of initial needle placement. A second line was then drawn from the medial canthus along the lamina papyracea (medial orbital wall, MOW) and the angle between the reference line and this line was recorded (Fig. 2). A third line was drawn from the medial canthus tangential to the medial wall of the globe without traversing the globe at any point, and the angle between this third line and the reference line was recorded. The length of the third line from the medial canthus to the optic nerve sheath was also recorded (Fig. 3). A fourth line measuring 25 mm was drawn to attempt to contact the medial aspect of the optic nerve sheath without traversing the globe. If this was possible, the angle between the reference line and the fourth line was measured and recorded (Fig. 4). The globe diameter was measured from anterior to posterior, from the cornea to the inner wall of the posterior globe (Fig. 5). This measure is a metric similar to the axial length measured for cataract surgery by partial coherence interferometry. Finally, a safe orbital space angle was defined as the difference between the angle between the reference line and globe tangent and the angle between the reference line and the medial orbital wall, to give an indication of the range of safe entry angles when delivering a medial canthal peribulbar block.

### Inter-observer variability

As the data was collected by two observers, inter-observer variability was addressed by calibrating the measurements of the initial 15 patients. A margin of +/− 5 degrees for the angle measurements and margin of +/− 2 mm were accepted for the purpose of this study. The observers repeated the calibration process for the 15 patients until the inter-observer variability was within the acceptable margin. After the calibration process, all patients fitted the pre-determined margin of variability.

### Statistical analysis

All data analysis was performed with MATLAB (The MathWorks, Inc., Natick, MA). Two sample t-tests were performed to investigate differences in metrics due to laterality or gender. Pearson correlation coefficients were determined to look for relationships between the measurements, with significance considered where *p* < 0.05. The distribution of parameters was investigated to identify orbits where the size might have an impact on clinical risk of iatrogenic injury.

## Results

In total 200 CT scans were analysed, consisting of 50 each of male and female right and left orbits. The average measurements are shown in Table 1.

**Table 1:** Average measurements of sample

	Average (SD)	Number	Comment
Age (years)	74.25 (9.50)	200	
Axial length (mm)	23.79 (1.02)	200	
Angle to globe tangent (degrees)	25.79 (7.68)	200	
Angle to optic nerve	24.85 (5.82)	81	For orbits where MC to ON distance < 25 mm
Angle to medial orbital wall (degrees)	6.68 (4.48)	76	For orbits where MOW lateral to reference line
Minimum distance between MC and ON (mm)	26.22 (3.76)	200	

Significant association was found between gender and the angle to the globe tangent, with the angle greater in male orbits (mean (standard deviation) in males = 27.65 (7.69) degrees, females = 23.93 (7.24) degrees, *p* = 0.000535). Conversely, the minimum distance between the medial canthus and the optic nerve was shorter on average in male compared to female orbits (male = 25.68 (3.83) mm and female = 26.75 (3.63) mm, *p* = 0.04). There was a strong negative correlation between orbital space angle and the minimum distance to the optic nerve (*r* = − 0.62, *p* < 0.0005). There was a weak correlation between increasing age and the angle of lateral deviation of the MOW (*r* = 0.15, *p* = 0.033). There was a significant but weakly negative correlation between the angle of intrusion of the MOW and the minimum medial canthus to optic nerve distance (*r* = − 0.22, *p* = 0.002).

There were no significant differences in any of the measurements between right and left orbits (*p* values 0.45–0.82). There was no significant correlation between axial length and any of the other measurements taken here.

### Distribution of data analysis

**Fig. 6:**
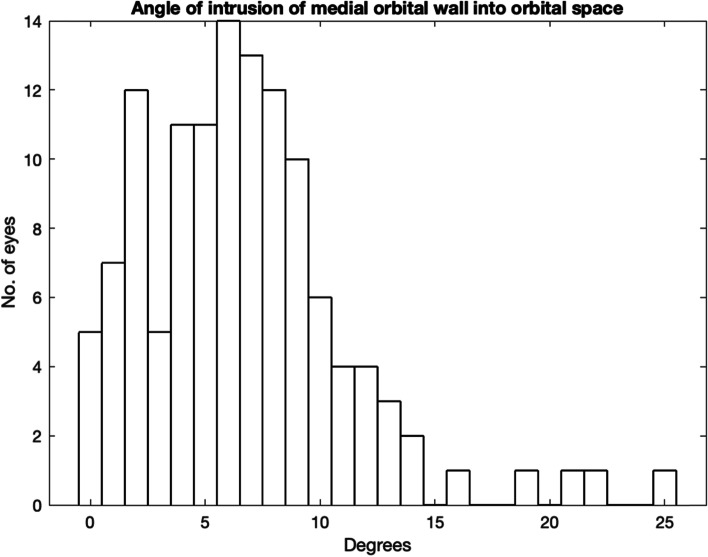
Angle of intrusion of medial orbital wall into orbital space

In 38% eyes the MOW intrudes into the orbital space as shown in Fig. 6, with a maximum of 25 degrees.

**Fig. 7:**
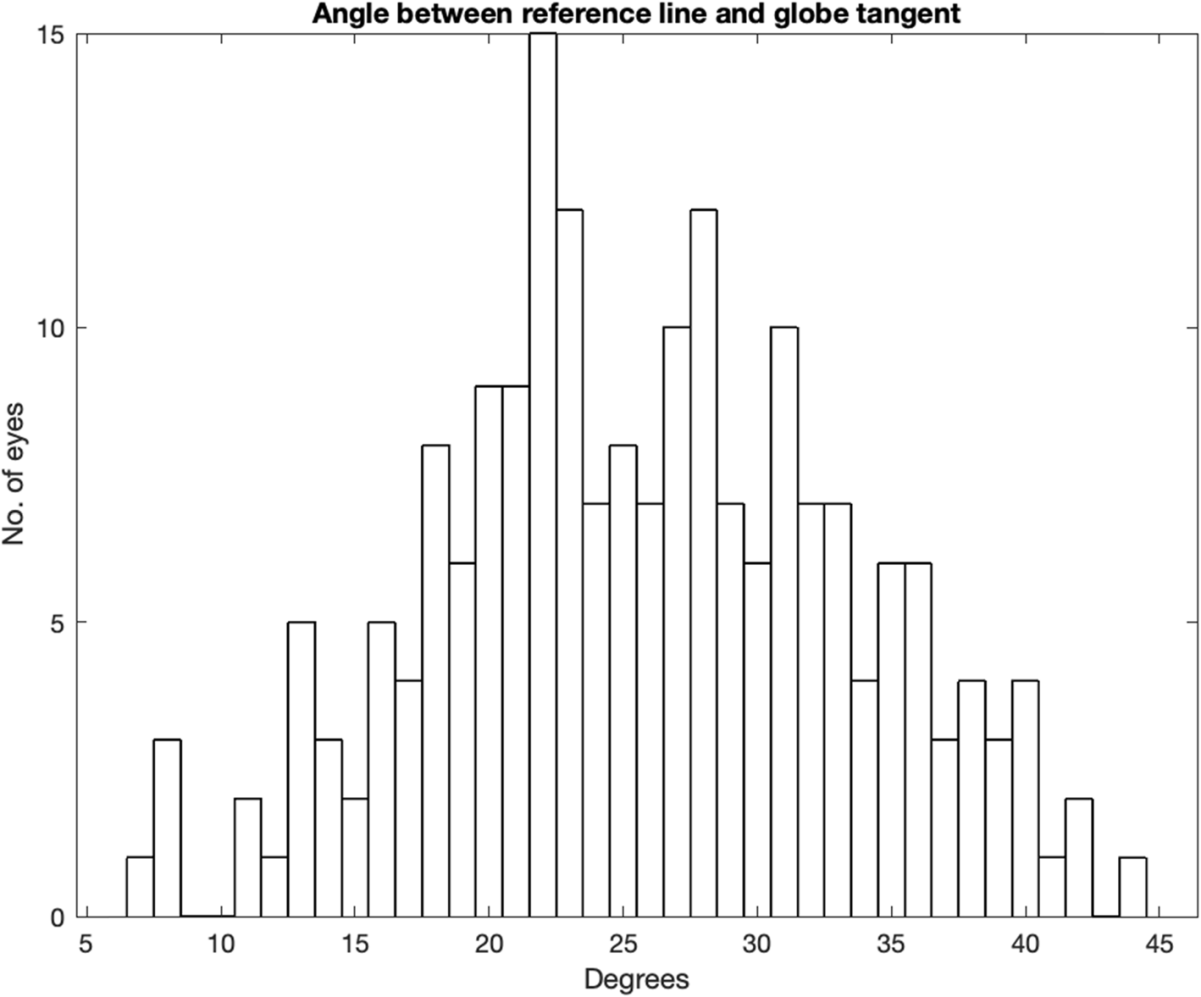
Angle between reference line and globe tangent

Figure 7 shows the angle from the reference line to the point where the optic nerve was 25 mm from the medial canthus. There are a total of 81 orbits (40.5%), and in 16/81 orbits (~ 20%) the angle is less than 20 degrees with 3 cases of only 14 degrees.

**Fig. 8:**
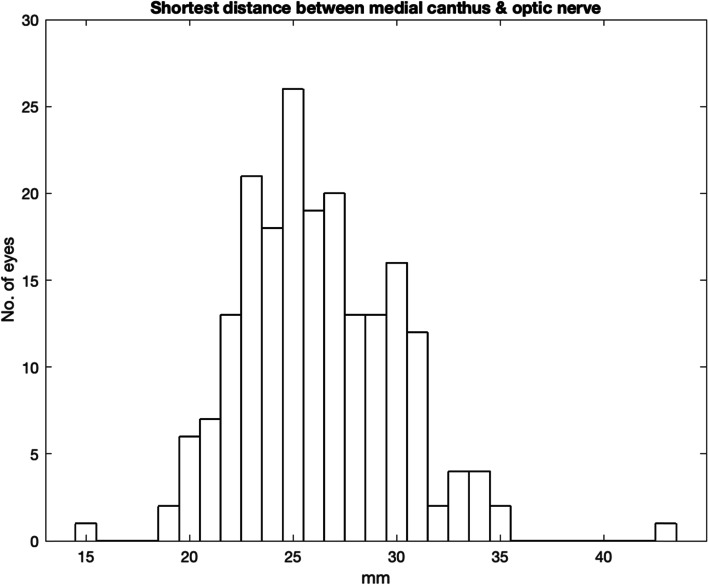
Shortest distance between medial canthus and optic nerve

Figure 8 shows the angle between the reference line and the globe tangent representing the safe space for needle insertion. 4 cases have an angle of less than 10 degrees. These 4 cases have an angles of intrusion of between 0 and 4 degrees.

**Fig. 9:**
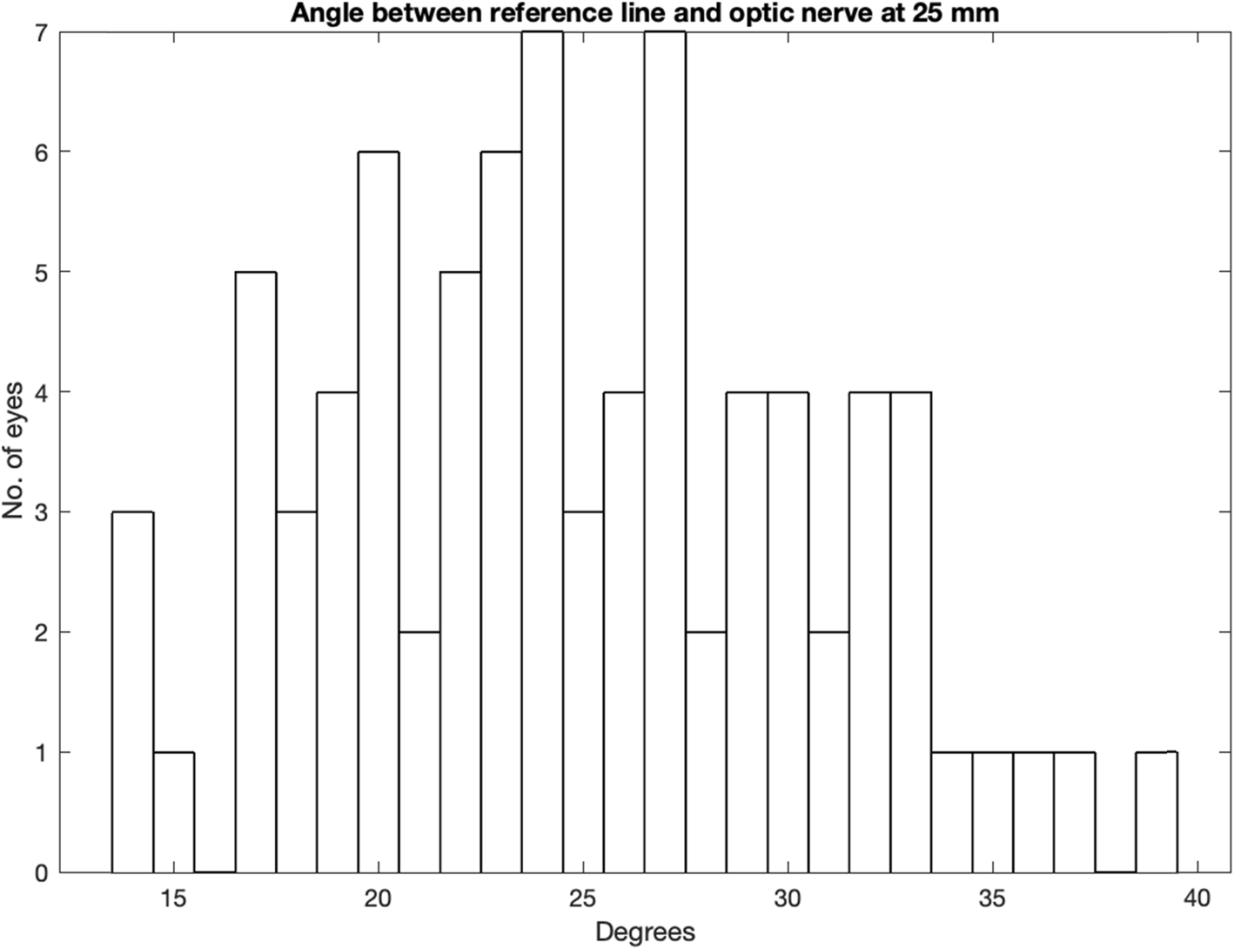
Angle between reference line and optic nerve at 25 mm

As shown in Fig. 9, 3 cases (1.5%) have a medial canthus to optic nerve distance less than 20 mm and a single case had a distance of only 15 mm.

## Discussion

This study used CT scan imaging to assess two of the risks of inserting a 25 mm needle into the medial canthus for peribulbar anaesthesia. First, penetrating the optic nerve sheath and second, globe perforation.

### Optic nerve sheath perforation

This study found that a 25 mm needle could reach the optic nerve sheath in 40.5% of the sample. In 1.5% the distance was 20 mm or less. Obviously, the risk would increase with a lateral angulation of the needle. These results show that with a needle angulation of 20 degrees or less from the reference line, 8% of orbits have the optic sheath within 25 mm. The risk of optic nerve injury may actually be increased in the clinical situation. In our study and in the ideal performance of medial peribulbar block, the eye is in a neutral gaze. I If the patient has lateral gaze (away from needle insertion), then the optic nerve is more exposed [14]. In addition, local anaesthesia, injected under pressure, close to but outside the optic nerve sheath may still cause brainstem anaesthesia [15]. Fortunately, published incidence of brainstem anaesthesia and optic nerve is an order of magnitude lower than 1.5%. The distance between the medial canthus to the optic nerve along the globe tangent is 25 mm or less in 49% of the sample, but there was one outlying case, where the distance was only 15 mm. This represents anatomy that is at high risk for this complication.

### Globe perforation

It would seem reasonable that as the safe space of the angle between bone and globe decreases, risk must increase. However, what angle may be considered acceptable is unclear.

Risks of globe perforation are already known to be increased in certain situations. Firstly, in those myopic patients with increased axial length. This is thought to be related to a larger diameter globe occupying more of the orbit and irregularities of globe shape including staphyloma [16, 17]. In this study those with a longer axial length did not have a reduced reference line to globe tangent angle. Although the single CT slice analysis means globe irregularities above and below this slice would be missed. However, staphyloma are rare on the medial aspect of the globe. A published incidence is of 82% inferior, 18% posterior and 0% medial [9].

Secondly, some techniques of peribulbar anaesthesia use 2 sites of injection, usually infero-temporal and medial canthus. There is an increased risk with the second injection. This is because the volume of the first injectate moves the globe toward the site of the second injection [18]. The bone-globe safe space is acutely reduced.

In this study, the average angle between reference line and globe was 26 degrees, with an average reduction due to the nasal bone of 7 degrees. This would seem to provide a reasonable safe space for needle placement in the average patient. However, when we look at the extremes of the study population, there were 2 patients where the available angle was only 9 and 10 degrees. There was a patient where the angle of bone intrusion was 25 degrees. Safe anaesthetic delivery must be reduced in these cases.

The measurements taken in this study indicate a continuum of risk. We were unable to correlate any of the routine pre-operative measurements (age, gender, axial length) to reduced intra-orbital measurements that increase risk. However, we did show a negative correlation between medial canthus to optic nerve distance and the reference line to globe angle. Thus, as the risk of globe perforation increases due to a narrow safe space, the risk of optic nerve injury decreases and vice versa. This may be associated with the degree of exophthalmos, but we did not assess this.

The negative correlation between orbital space angle and the minimum distance to the optic nerve means that as the safe angle of entry increases the distance to the optic nerve falls. This is likely due to the larger angle to the globe tangent reflecting a more anteriorly positioned eyeball, rather than posterior migration of the medial canthus.

The association between gender and the angle to the globe tangent reflects the larger skull size of men compared to women. However, the average distance between the medial canthus and optic nerve was lower in male orbits compared to female. This is an unusual finding as interpupillary distance is typically lower in females compared to males [19]. As the angle to the optic nerve was larger in males, this suggests the optic nerve (or back of globe) is more anteriorly positioned in females. There is a lack of any correlation between axial length and these measurements. This means that the proceduralist has, at the time of anaesthetic, no guide as to how to safely perform a medial canthal peribulbar block.

To reduce the risk of complications, these patients need to be identified by other means such as ultrasound imaging. Alternatively, use of needle blocks could be reduced by utilising existing safer techniques [1, 3, 4, 10].

Our teaching of the peribulbar block to minimise complications is to keep the needle perpendicular. Only deviating laterally if bony resistance is encountered and by the minimal angle. Do not insert the full 25 mm length, or consider using a 16 mm needle. These “rules of thumb” are supported by our results.

One inference from this study may be to walk the needle along the bone of the medial orbital wall. The disadvantage is that many older patients have bone that is extremely thin. This is easily penetrated giving an erroneous needle placement.

We accept that a complication of superficial needle placement is an inadequate block. Therefore the depth used is a compromise of clinical factors in the individual situation.

We believe that a single medial injection is the safest technique. This study shows that there are patients where imaging the orbit could potentially increase this still further. Therefore, we can speculate on possible future issues from this. The orbit and its contents, that we used in this CT study, are easily imaged with ultrasound. Given the increasing usage and expertise in many areas of anaesthesia coupled with litigation risks, it may become a standard of care to at least view the orbit if not actually performing a real time imaging. This then has issues if imaging is unavailable. The current culture of safety is not yet present at this level.

There are some limitations to the study. Firstly, the demographic and clinical data available was very limited. There may be factors not studied with predictive value such as ethnicity. Secondly, whilst the study provided adequate data to sample the general population, and demonstrated the presence of higher risk cases. The numbers of these higher risk cases were too few for further analysis.

We have shown that there are a minority of patients with unusual orbital anatomy. This places them at a theoretical higher risk of complications. These cases are not currently predicted by current pre-operative information.

We hope this study gives a better appreciation of risk for those performing medial peribulbar anaesthesia and will stimulate further work to identify those patients with increased risk.

## Data Availability

The raw data used in this study is available on request from the corresponding author.

## Abbreviations

CT: Computed Tomography
HU: Hountsfield Units
MPR: Multiplanar reconstruction
MOW: Medial Orbital Wall
RL: Reference Line
